# Electronic Nose with Digital Gas Sensors Connected via Bluetooth to a Smartphone for Air Quality Measurements

**DOI:** 10.3390/s20030786

**Published:** 2020-01-31

**Authors:** Patricia Arroyo, Félix Meléndez, José Ignacio Suárez, José Luis Herrero, Sergio Rodríguez, Jesús Lozano

**Affiliations:** Industrial Engineering School, University of Extremadura, 06006 Badajoz, Spain; parroyoz@unex.es (P.A.); felixmv@unex.es (F.M.); jmarcelo@unex.es (J.I.S.); jherrero@unex.es (J.L.H.); sergiorodriguez@unex.es (S.R.)

**Keywords:** electronic nose, gas sensors, smartphones, air quality, metal oxide

## Abstract

This paper introduces a miniaturized personal electronic nose (39 mm × 33 mm), which is managed through an app developed on a smartphone. The electronic nose (e-nose) incorporates four new generation digital gas sensors. These MOx-type sensors incorporate a microcontroller in the same package, being also smaller than the previous generation. This makes it easier to integrate them into the electronics and improves their performance. In this research, the application of the device is focused on the detection of atmospheric pollutants in order to complement the information provided by the reference stations. To validate the system, it has been tested with different concentrations of NOx including some tests specifically developed to study the behavior of the device in different humidity conditions. Finally, a mobile application has been developed to provide classification services. In this regard, a neural network has been developed, trained, and integrated into a smartphone to process the information retrieved from e-nose devices.

## 1. Introduction

Most research studies and media emphasize that air pollution is one of the greatest environmental health risks worldwide. Nevertheless, it is often imperceptible to people, which makes it go unnoticed. According to the latest data from the World Health Organization (WHO), air pollution causes up to 7 million deaths a year [1]. In addition, there are numerous research studies demonstrating the adverse effects of pollution on almost every system of the human body [2,3,4]. However, although the poor quality of air is widespread, it is invisible to the naked eye, and therefore people are not really aware of the levels of air quality to which they are exposed at every time and place. Normally, reference air quality measurement systems are found in a few locations. It is generally due to the high cost of air quality monitoring stations, which makes it difficult to get real-time relevant data with sufficient spatial resolution.

Consequently, several organizations and countries worldwide have established air quality monitoring systems. Therefore, this approach requires a high budget, which in general implies an insufficient number of measurement points. However, these systems have a high cost and size, resulting in an insufficient number of measurement points. Furthermore, the location of air quality stations is not always sufficiently representative. Therefore, there is a need for small, low-power devices for personal air quality monitoring. As a result, different small, portable, and low-power devices have emerged [5,6,7,8,9,10]. While these devices are less accurate and show a higher uncertainty compared with reference systems, they increase the spatial density of measurements and could provide useful information to citizens. Namely, they are not a replacement for reference instruments, especially for official purposes, but rather they are complementary sources of air quality information [11].

The most efficient solution to obtain measurements of a high number of locations is by means of wireless sensor networks (WSN) [12]. With the increasing acquisition of smart personal mobile devices with Internet connection, such as smartphones, it is interesting to consider their incorporation into this kind of system. In this way, each mobile device user could be viewed as a sensor node integrated in a wireless network. This would create a higher resolution air quality indicative map. For this purpose, the air quality measurement device must be small, so that it can be easily carried and does not cause any inconvenience to the holder or citizen. In this regard, Mobile Sensing Systems (MSS) have emerged, which are mobile sensor systems made up of smartphones (to control the sensors), a web server to store data, and the use of protocols and cloud computing to send and retrieve the data. These systems have been reviewed by several authors [13,14,15].

In the field of gas sensors suitable for electronic noses (e-noses), there are five main types: resistive, surface acoustic wave, catalytic, optical, and electrochemical [16]. According to the literature [17], electrochemical gas sensors exhibit better behavior in comparison with the reference methods; however, resistive gas sensors are the most commonly used in portable and miniaturized devices due to their small size and low power consumption, in particular those based on semiconductor metal oxides (MOx). They also offer fast response and recovery times at very low costs. In addition, thanks to the technology of Microelectromechanical Systems (MEMS), these sensors have drastically reduced their dimensions, reaching sizes of up to 3 × 3 mm. As a consequence, power consumption has also been reduced considerably. The MOx sensors are made up of a metal oxide film or filament (usually tin dioxide) doped with other compounds such as tungsten trioxide or zinc oxide (type n) or nickel oxide (type p). When it comes into contact with the volatiles in the air, an electronic depletion layer is formed, resulting in a change in the conductivity of the sensors [18]. In recent years, miniaturized MOx sensors have emerged with a processing unit integrated in the same package. These sensors include the electronic reading (reducing electrical noise derived from external conditioning circuits), a preprocessing (manufacturer’s algorithms), and also provide the readout via a digital output (I^2^C/SPI). Moreover, in some cases, temperature, humidity, and pressure sensors are integrated besides the gas sensor. These sensors are of great novelty and interest in personal devices such as those that are intended to develop in this work. However, nowadays, there are still few studies in the bibliography that include them. Some of these sensors have previously been used to monitoring plants activity [19], to study their individual response to BTEX (Benzene, Toluene, Ethylbenzene and Xylenes) compounds [20], or to investigate their architecture and operation (specifically Sensirion’s SGP30 model) [21].

Among the main atmospheric pollutants are nitrogen oxides (NOx). These oxides are formed due to the oxidation suffered by atmospheric nitrogen (N_2_), the main component of air, at high temperatures. The term NOx is commonly used to designate the nitrogen oxides that affect air pollution: nitric oxide (NO) and nitrogen dioxide (NO_2_). These gases are the principal agents of smog, acid rain, and precursors of the tropospheric ozone. It is worth noting that part of the NOx emissions is due to natural causes (bacterial decomposition of organic nitrogen, forest fires, volcanic activity, storms, etc.). However, the biggest source of emissions is from anthropogenic causes: the use of fossil fuels and exhaust from combustion vehicles [22].

The miniaturized electronic nose presented in this paper integrates four of these processing-capable MOx sensors. The device is small sized in order to be easily portable, and each user can be a network node. It communicates wirelessly with an intelligent device (smartphone) and is controlled through an app developed in Android. Since MOx sensors react to a high number of compounds, it is necessary to apply pattern recognition techniques for increasing the selectivity of the detection system to the target compounds. There are several techniques that have been used previously and reviewed in the literature dealing with applications for electronic noses [23,24,25]. For this case, it was decided to program and train a neural network (NN) integrated into a smartphone to manage and monitor the electronic nose. It allows fast data processing and a decrease in the flow of data to be sent and received from the smartphone.

The complete system is described in detail throughout the paper. First, the design of the electronic nose is described, focusing on the included sensors. The following part deals with the development of the mobile application in charge of processing the data retrieved from the e-nose device. In order to study the detection and discrimination capacity of the device, some laboratory tests have been performed. These experiments have been carried out with different concentrations of NOx, which is one of the main contributors to air pollution. The measurement procedure and the obtained results are presented and discussed in Section 3. Finally, the conclusions are presented.

## 2. Materials and Methods

### 2.1. Electronic Nose Description

**Figure 1:**
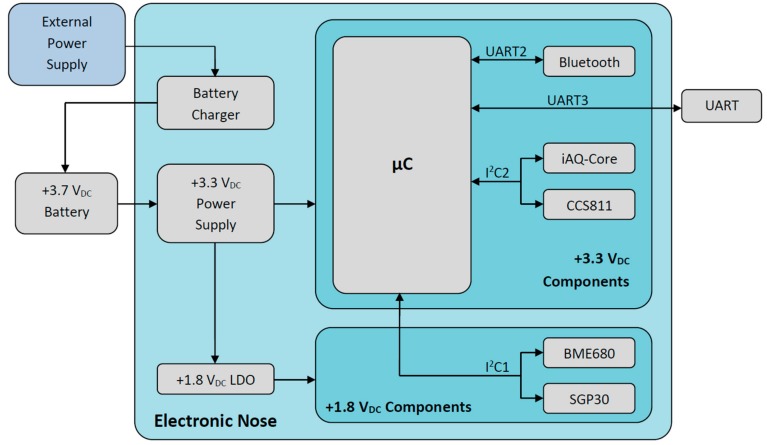
Block diagram of the developed electronic nose.

The block diagram of the developed electronic nose prototype is depicted in Figure 1. The power source is composed of a battery charger, a +3.7 VDC Li-ion battery, a +3.3 V dc-dc converter, and a +1.8 V low drop-out linear regulator.

The core of the system is a 32-bit microcontroller, model PIC32MM0256GPM048 from Microchip, which performs the main operations: sensor control through two I2C serial interfaces; communications with smart devices by using a Bluetooth low-energy RN4871 tiny module from Microchip; and wired UART communications with other devices. This microcontroller has been chosen because of its low current consumption thanks to its low operating frequency (24 MHz). Despite of that, it has a great processing power due to its 32-bit architecture, which is complemented with a 256 KB program memory and a 32 KB data memory.

Four digital gas sensor chips have been installed. However, due to their different supply voltages (+1.8 V and +3.3 V), it has been decided to use two separate I^2^C interfaces to achieve more simplicity. 

Regarding the power consumption of the electronic nose, a theoretical maximum current (according to manufacturers) of 412 mA has been calculated, although the operating current measured experimentally was up to 185 mA.

**Figure 2:**
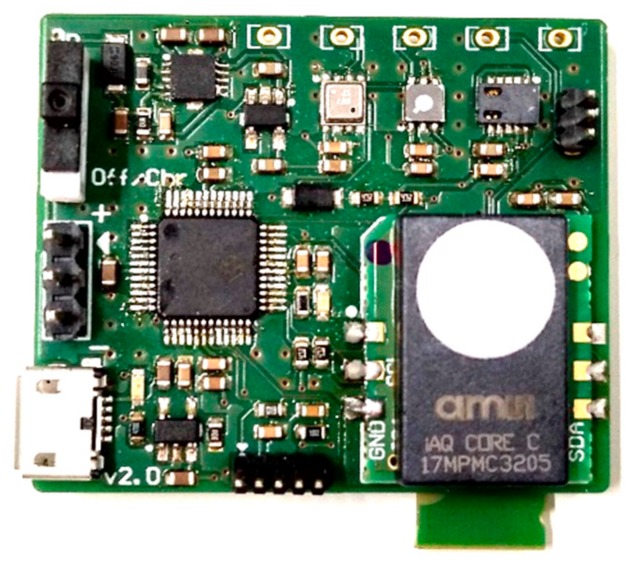
Developed electronic nose.

In Figure 2, a picture of the developed electronic nose is shown. The dimensions of the device are 39 mm × 33 mm.

### 2.2. Gas Sensors

**Figure 3:**
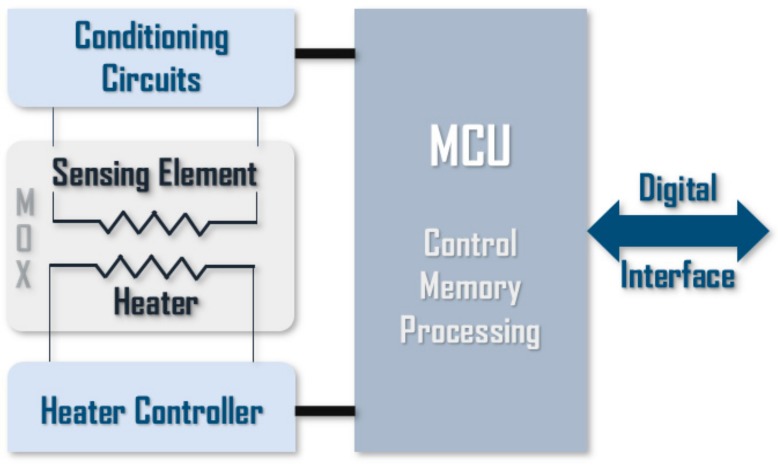
General block diagram of digital gas sensors.

The four chips, which integrate metal oxide (MOX) sensors, are the following: BME680 from Bosch [26], SGP30 from Sensirion [27], and CCS811 [28] and iAQ-Core [29] both from AMS. All of them are miniaturized intelligent gas sensors. These devices are characterized by the integration of analog and digital electronics combined with a hot microplate and the detecting elements on a single chip. In general, the signals coming from the resistive sensing elements are received by the processor through conditioning analog circuits. Then, these signals are processed with different algorithms (averaging, baseline compensation, humidity correction, etc.). Furthermore, some of them allow users to write calibration parameters. Finally, the output signals (calibrated and/or raw) are transmitted using digital communication protocols. This integrated digital interface greatly simplifies the integration of these sensors into the electronic nose. Figure 3 shows a block diagram of the main components and functionalities of digital gas sensors. 

The main characteristics of the gas sensors are indicated in Table 1. All sensors, except BME680, include intelligent algorithms to process the raw signals to output TVOC (Total Volatile Organic Compounds) and equivalent CO_2_ (eCO_2_) prediction values. Additionally, SGP30 provides raw signals for H_2_ and ethanol.

The BME680 also includes temperature, relative humidity, and atmospheric pressure sensors, whose ranges are −40 to +85 °C, 0% to 100%, and 300 to 1100 hPa, respectively.

### 2.3. Communication Protocol

A simple ASCII-based protocol has been established for the Bluetooth communication between the microcontroller and an external smart device. There are some commands for retrieving data from individual sensors and some for changing sensor parameters, setting heaters values, etc. Every sample time, the microcontroller reads sensor values and sends them via Bluetooth in a formatted frame. Each data frame is composed of columns separated by horizontal tabs and finished with carry return and line feed characters. The meaning of each column can be found in Table 2.

The 16th column shows a 0 when the electronic nose is smelling clean air (desorption), and it shows a 1 when is smelling a sample (absorption). The 17th column is used to notify the user (who has an app in the smart device) when to manually switch from a desorption phase to absorption phase or vice versa.

### 2.4. App and Neural Network

A software tool (app) has been developed for processing the data provided by the electronic nose. The following requirements have been considered:

1. Portability: As the small size and light weight are some of the main advantages of the e-nose device, it is most suitable for outdoor environments. To get more benefits from these advantages, this work has considered mobile devices as the target hardware where a software application should be install to monitor and control e-nose data.
2. Connectivity: The tool must support connection with the e-nose device and it is also required to implement the correct communication protocol to interchange data between both systems.
3. Data processing: Sensor data need to be processed before they can be used. In this regard, the tool should apply data processing algorithms.
4. Data classification: A data classification algorithm must be applied to categorize sensor data and provide useful information for the users.
5. Performance: The tool must receive sensor data in short intervals of time while processing and classifying the information without affecting the overall system operation.
6. Response time: User requests must be processed and served immediately.

To deal with all this specifications, first, Android has been selected as the mobile operating system, because its great advantage is that it is widely spread over millions of devices around the world. A Bluetooth low-energy communication module has been selected to interconnect both systems (e-nose device and the smartphone) because of the long autonomy that this alternative provides. Next, data normalization and feature extraction algorithms have been applied. Finally, machine learning algorithms have been selected as the best approach to classify data retrieved from the e-nose. In this case, neural networks have been applied, since performance and short response times are achieved once the neural network has been previously trained. 

**Figure 4:**
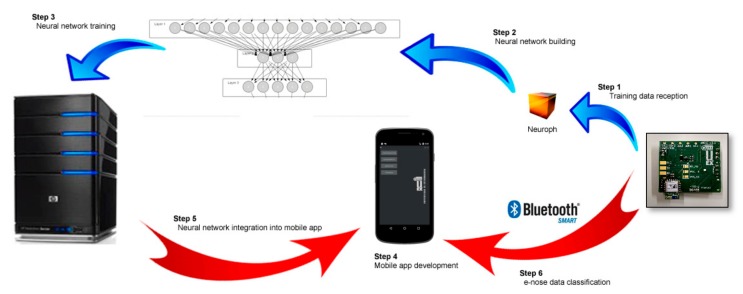
Methodology followed to develop the mobile application.

According to system requirements, different technologies can be applied for software development. Cloud-based approaches offer a technology that provides magnificent elastic computation and data management abilities for the Internet of Things (IoT). This approach is aimed at formulating a complex information system with the combination of sensor data acquisition [30]. A previous work [31] presented a cloud-based proposal to integrate services for e-nose devices. However, this approach was more appropriate when short response times were not required and connectivity was always guaranteed. In scenarios where response time is a priority and/or connectivity cannot be achieved, this approach is not applicable and different alternatives must be addressed, which is the scenario on which this work focuses. To deal with this problem, this section proposes the integration of the processing and classifying algorithms into smartphones. Figure 4 shows the methodology followed for developing the proposed tool, which is divided into the following steps.

1. Neural network building: The aim of this level is building and training a neural network using e-nose data extracted from a set of experiments. As smartphones do not provide enough performance to process all the information, a high capacity computer has been chosen for neural network training. In this level, Neuroph software has been used to build and train a multilayer neural network composed by three layers with 10 inputs (one for each sensor signal) and nine outputs (one for each compound classified). This is an open source tool for machine learning development and has been selected among others because it provides specific libraries to connect mobile applications with neural networks.
2. Mobile application development: A mobile app has been implemented to monitor and process all the information retrieved from the e-nose device. The Android Studio tool has been used for developing this software and Neuroph libraries have been integrated in order to manage the neural network. The functionality of this tool is diverse: from connecting with the external device through Bluetooth low energy to applying classification algorithms to categorizing sensor data, passing by the processing and the storing of the data for later analysis.

## 3. Results

In order to study the performance of the electronic nose for detecting and discriminating contaminants, some measurements have been made in the laboratory. Specifically, NO and NO_2_ have been measured at different concentrations.

### 3.1. Measurements Set-Up

**Figure 5:**
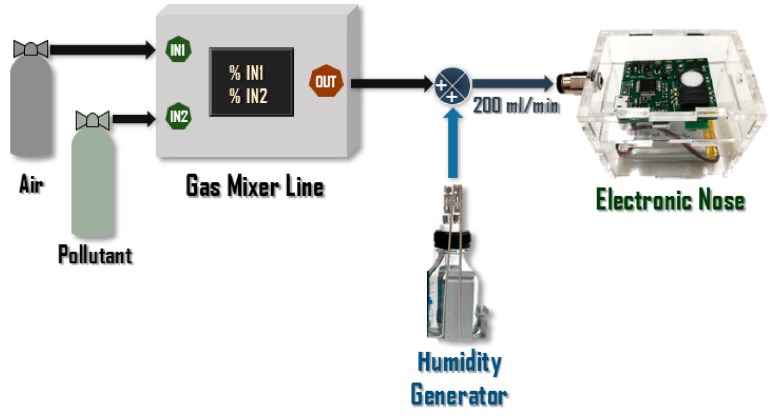
Measurements setup.

A homemade gas line has been developed and used to perform pulses of NO_2_ and NO at different concentrations. This system (Figure 5) allows to mix up to four gases from gas cylinders at the desired concentration. It is composed by mass flow controllers, electrovalves, and a gas expansion module, and it is controlled by a PLC (programmable logic controller) with a touch screen. The gas line allows programming customized and timed cycles to generate gas mixtures. In this specific case, 10 measurement cycles have been programmed for each concentration, in which dry air is delivered to the sensors for 4 min and a mix of the pollutant to be measured at the desired concentration with dry air is generated for 2.5 min. At the output, it is mixed with an adjustable humidity flow, which is also taken into account when calculating the desired concentration. During the whole experiment, the relative humidity was 0%. The flow rate to the electronic nose was always fixed to 200 mL/min.

The used cylinders have an initial composition of 0.81 molppm in the case of NO_2_ and 125 ppbvol in the case of NO. The concentrations generated for NO_2_ are 40 µg/m^3^, 80 µg/m^3^, 120 µg/m^3^, 165 µg/m^3^, and 200 µg/m^3^. With regard to NO, four different concentrations have been generated: 7.7 µg/m^3^, 15.5 µg/m^3^, 38.5 µg/m^3^, and 77 µg/m^3^. It is remarkable that the concentrations chosen for the case of NO_2_ are governed by the official 1-h exposure limit of 200 µg/m^3^ according to the air quality standards of the European Union [32]. Since there are no standards for NO because it is a precursor, low concentrations easily generated by the system have been chosen.

On the other hand, humidity tests have been carried out with the purpose of studying how this could affect the detection capability of the device and to be able to take future precautions. Variations in humidity have been measured in the same way as described above. Throughout, 40 µg/m^3^ of NO_2_ has been generated in cycles of 4 and 2.5 min. Five different relative humidities have been produced: 0%, 10%, 40%, 70%, and 90%. As in the previous test, 10 measurement cycles have been carried out for each relative humidity generated.

To carry out these tests with gas cylinders, a watertight methacrylate housing has been designed with two pneumatic couplings that allow the gases to flow through the electronic nose. The volume corresponding to the cell through which the gas flows is 33 cm^3^ (with dimensions of 4 × 5.5 × 1.5 cm). The housing also includes an insulated bottom compartment to accommodate the battery. It is worth pointing out that this chamber and the calibration process will only be used for the laboratory tests carried out in this study in order to obtain an initial evaluation of the performance of the system. In future work, additional calibrations will be applied for the field tests in real conditions that are scheduled to be performed near reference stations for correlation tasks.

### 3.2. Pollutant Discrimination Results

Once the electronic nose takes measurements, the sensor signals are received in the smartphone. These signals are those specified between the fifth and 15th column listed in Table 2. However, the signal from SGP30 relating to eCO_2_ has been discarded because this signal has a minimum of 400 ppm that is never exceeded throughout the experiments. Therefore, the signals that are considered in these experiments will be named from now on as follows: BME680, SGP30_1 (H_2_ in Table 2), SGP30_2 (ethanol in Table 2), SGP30_TVOC, CCS811, CCS811_TVOC, CCS811_CO2, iAQCore, iAQCore_TVOC, and iAQCore_CO2.

Before data processing, it is necessary to pre-process the signals in order to obtain a characteristic value for each measurement cycle. Therefore, to perform this feature extraction, a baseline manipulation algorithm has been used. In the selected algorithm in this study, the obtained characteristic value is proportional to the difference between the baseline value (corresponding to the dry air flow) and the value reached during the pollutant measurement phase:

(*Vref* − *Vp*) × 100 − 1, (1)

where *Vref* is the value obtained from the sensor during the air measurement phase and *Vp* is the value in the pollutant measurement phase.

As a result, after having made all the measurements, a matrix of 10 (sensor signals) by 90 (10 cycles multiplied by nine NOx concentrations) will be obtained. Nevertheless, the first value of each concentration of each measured compound is discarded. Therefore, the final matrix will be 9 × 81.

**Figure 6:**
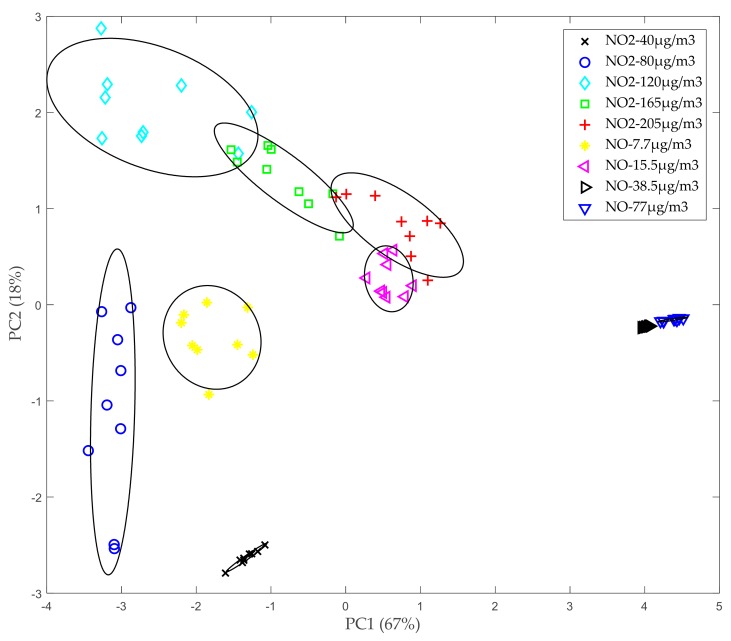
Principal component analysis (PCA) plot.

Subsequently, a reduction in dimensionality has been carried out using the Principal Component Analysis (PCA) [33]. This technique can describe a dataset in terms of new non-correlated variables (“components”). Components are sorted by the amount of original variance they represent, so the technique is useful for reducing the dimensionality and the redundancy of the dataset. In this way, just the first two main components (which will contain most of the information) can be represented in a plot to observe the distribution of the data. The resulting plot is shown in Figure 6. In this case, the first main component contains 67% of the information and the second one contains the remaining 18%. In general, it can be noticed that the different clusters can be differentiated, although there is overlap between zones such as NO_2_ 120 µg/m^3^, NO 165 µg/m^3^, NO_2_ 205 µg/m^3^, and NO 15.5 µg/m^3^. In some areas, such as NO_2_ at a concentration of 80 µg/m^3^, drift problems appear. Such problems are probably related to an insufficient sensor stabilization time before performing these measurements.

**Figure 7:**
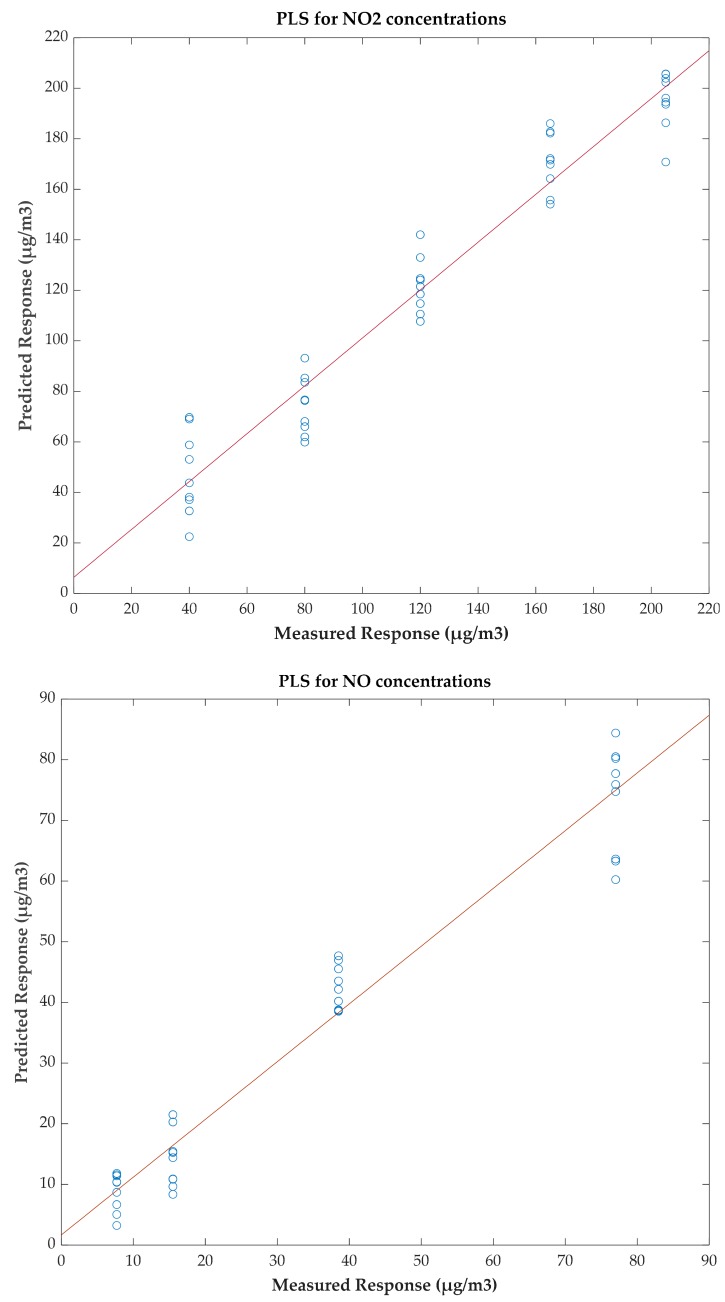
Partial Least Squares (PLS) plots.

On the other hand, correlation tests have been performed in order to evaluate the predictive capacity of the device in quantification tasks. For this purpose, a Partial Least Squares (PLS) regression has been performed and the predictions made by the model from the sensor responses have been represented (Figure 7). The actual value is represented on the X-axis, and the value estimated by the model is represented on the Y-axis. The coefficient of determination (R^2^) obtained is 0.947 for NO_2_ measurements and 0.951 for NO measurements.

Finally, for classification purposes, a neural network has been programmed into the smartphone app (externally trained). A multiperceptron architecture [34] has 10 neurons in the input layer, a hidden layer containing four neurons, and one neuron in the output layer. Afterwards, the LOOCV (leave-one-out cross-validation) validation technique was used to study the classification capability. Results are shown in the Confusion Matrix (Table 3). In this matrix, each column represents the predicted pollutant concentration and each row represents the real pollutant concentration. Consequently, the main diagonal contains correct predictions, while outside values correspond to erroneous predictions. Finally, a success rate of 92.59% is achieved in the classification, with six mistakes being committed out of 81.

**Figure 8:**
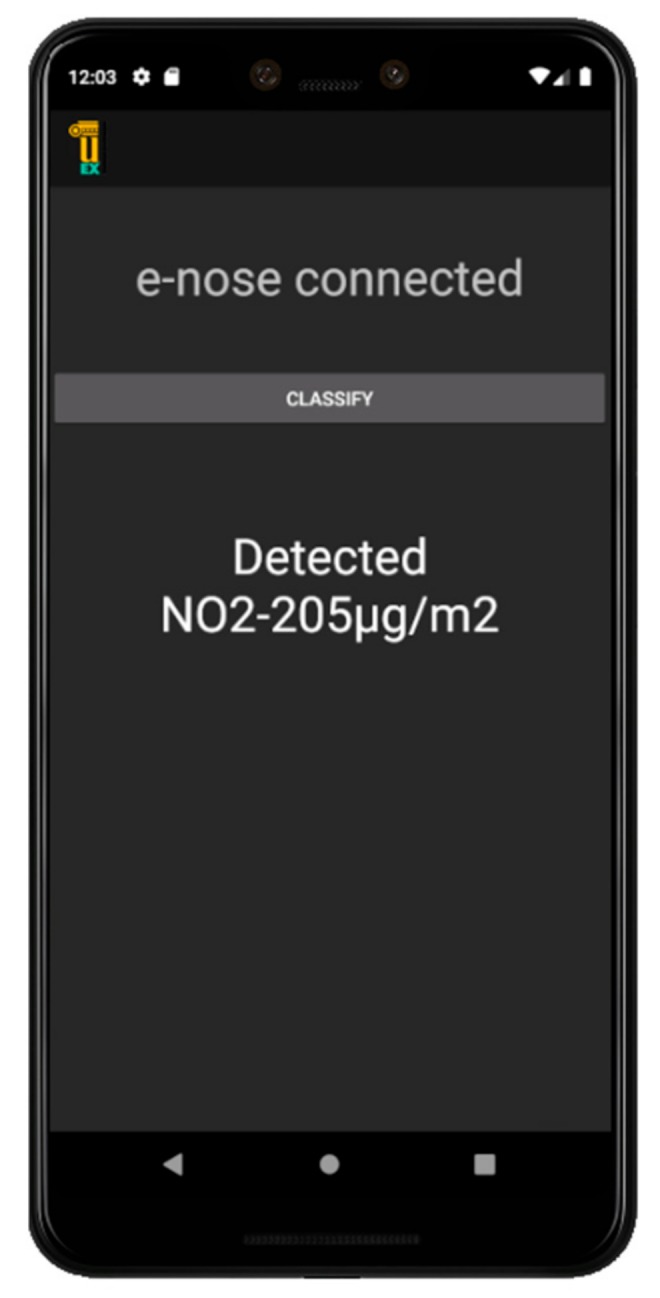
Smartphone screenshot classifying NO_2_ to a concentration of 205 µg/m^3^.

Figure 8 shows a screenshot of the classification result obtained on the smartphone app as an example.

### 3.3. Limit of Detection (LOD) Estimation

In addition, an estimation of the limit of detection of the electronic nose for NO and NO_2_ has been obtained. It has been calculated for each of the four sensors integrated in the device. For this purpose, the raw signal obtained from each sensor (Ω) is used, since all other signals are algorithms offered by the manufacturer that may be non-linear and in some cases have lower and upper limits implemented.

The estimated LOD (µg/m^3^) has been determined according to the simplified LOD formula [35,36]:

LOD = 3.3 × s_0_/Â, (2)

where Â is the slope of the resistive response of the sensor against the pollutant concentration and s_0_ is the standard deviation of blank measurements, i.e., measurements performed in clean air.

**Figure 9:**
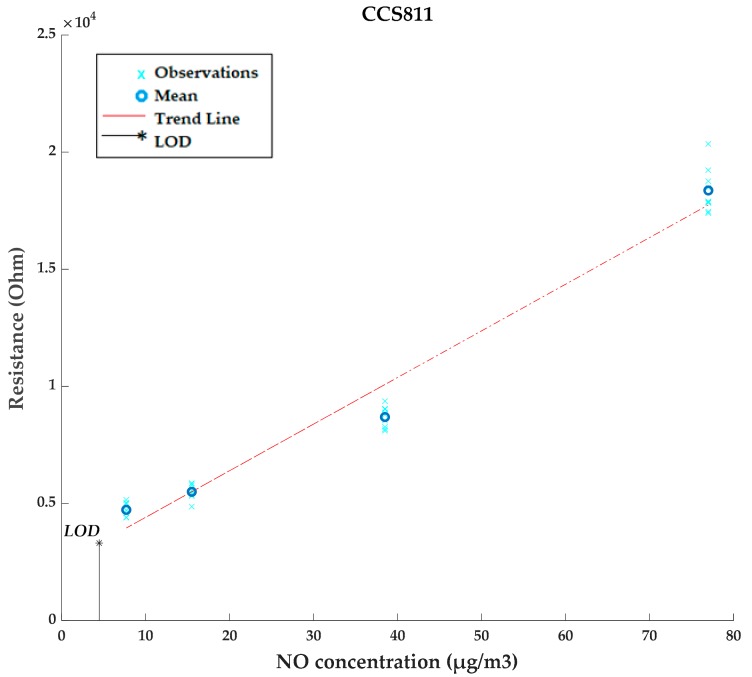
Measurements, trend line, and estimated limit of detection (LOD) of CCS811 for NO detection.

As an example, Figure 9 shows a chart with the data obtained by the CCS811 gas sensor for the different NO concentrations. The red line represents the trend line whose slope is used for the calculation of the LOD (Equation (2)), which is also represented in the figure (black line).

Table 4 summarizes the estimated NO_2_ and NO limits of detection for each sensor expressed in µg/m^3^.

### 3.4. Humidity Tests

**Figure 10:**
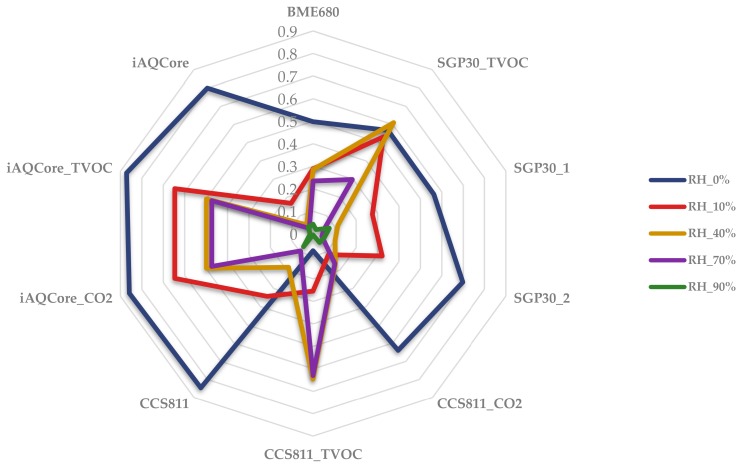
Radial diagram of the average responses of gas sensors at different relative humidity values.

NO_2_ measurements at a concentration of 40 µg/m^3^ have been performed under five different relative humidity conditions, with the aim of studying the response of the sensors under these different environmental conditions. The results have revealed that as the relative humidity increases, the response of the sensors decreases, or is even non-existent in this scenario. For each humidity value, 10 measurement cycles have been carried out. Figure 10 shows a radial graph of the averaged values of the 10 measurement cycles for each gas sensor. It can be verified that sensor responses are low for high values of relative humidity, except in some cases, such as the CCS811 TVOC response. This might be related to the internal algorithm implemented by the manufacturer.

**Figure 11:**
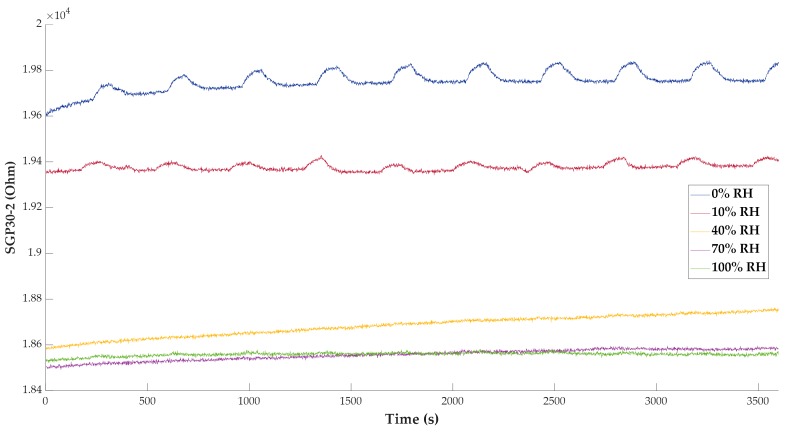
Time responses of sensor SGP30_2 (Ω) at different relative humidity values.

Sensor time responses can also be studied, as shown in Figure 11, where a comparative graph of the SGP30_2 response signals is depicted. It can be appreciated that below 40% of relative humidity, the responses are almost imperceptible.

**Figure 12:**
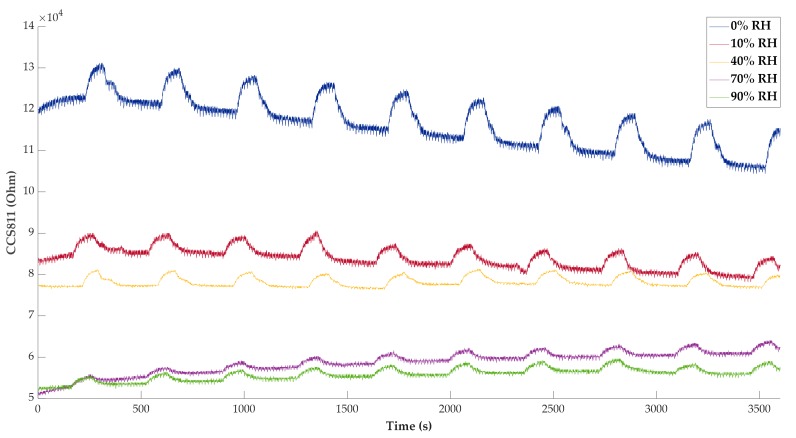
Time responses of sensor CCS811 (Ω) at different relative humidity values.

However, the operating range in this test is not the same for all installed sensors. Figure 12 depicts the resistive responses of the CCS811 sensor, which are, indeed, inversely proportional to relative humidity. Nevertheless, even at 90% relative humidity, a significant variation of the response to NO_2_ can be detected.

Therefore, a filtering unit or a humidity correction algorithm should be added in high humidity environments for the detection of low concentrations of pollutants. This must be taken into account for future improvements and developments.

## 4. Conclusions

In this paper, a low-cost, low-consumption, and very small size electronic nose for air quality monitoring has been illustrated. Typical gas sensors based on metal oxide technology that have been used in electronic noses for decades are not appropriate for miniaturized instruments due to their high power consumption and size. MEMS technology has allowed the integration of gas sensors within CMOS technology modules, which embed signal processing, A/D converter, and communication circuits. These digital sensors that have appeared in the market in the last years normally incorporate several gas sensors or even others such as atmospheric pressure, humidity, or ambient temperature sensors.

On the other hand, the use of miniaturized measurement devices of small size, low power consumption, and cost in combination with smartphones allows the creation of sensor networks in which each citizen acts as a node of a huge network with a very high number of nodes. This configuration, together with the great computation capacity of the processors of the latest telephones, makes it possible to execute complex signal processing techniques in the device itself without having to increase the amount of data sent or received.

The detection and discrimination capability of the device with different concentrations of NOx compounds (some of the main atmospheric pollutants) have been checked. For this purpose, a multiperceptron neuronal network has been programmed in the app developed for smartphones. It has been performed several tests with different conditions of relative humidity to study whether it affects the detection performance of the gas sensors. It was concluded that high humidity conditions could significantly affect the operation of the device for this application, especially in the detection of low pollution concentrations.

The results obtained indicate that significant future work will be required to improve the reliability of MOx sensors in the presence of humidity in order to obtain a good prediction in the concentration of pollutants in air. However, these digital sensors may be integrated in the near future in smart devices, such as phones, watches, or tablets, following the trend of increasing the number of sensors in these devices. This integration, and its massive use, will allow the appearance of numerous applications that will make use of the sensor data as it happened some years ago with the cameras integrated in the smartphones and the apps of artificial vision. Such prototypes and advanced algorithms for signal processing are already under development, as discussed, and will likely be required for the widespread and successful deployment of e-noses as personal devices for air quality monitoring.

## Figures and Tables

**Table 1:** Digital metal oxide (MOX) gas sensors main characteristics. TVOC: total VOC.

Sensor	BME680	SGP30	CCS811	iAQ-Core
Manufacturer	Bosch	Sensirion	AMS	AMS
Supply Voltage [V]	1.71 to 3.6	1.62 to 1.98	1.8 to 3.6	3.3
eCO_2_ range [ppm]	see note ^1^	400 to 60,000	400 to 29,206	450 to 2000
TVOC range [ppb]	see note ^1^	0 to 60,000	0 to 32,768	125 to 600
I^2^C Interface [kHz]	up to 3400	up to 400	up to 400	up to 100
SPI Interface [MHz]	up to 10	No	No	No
Size [mm]	3.0 × 3.0 × 0.93	2.45 × 2.45 × 0.9	2.7 × 4.0 × 1.1	15.24 × 17.78 × 4.3

^1^ BME680 provides a unique gas reading, which corresponds to sensor resistance in ohms.

**Table 2:** Column information for each data frame.

Column	Description	Sensor
1	Sample number	
2	Temperature [°C]	BME680
3	Pressure [hPa]	BME680
4	Humidity [% RH]	BME680
5	Gas measurement [Ω]	BME680
6	eCO_2_ [ppm]	SGP30
7	TVOC [ppb]	SGP30
8	H_2_ (see note ^1^)	SGP30
9	Ethanol (see note ^1^)	SGP30
10	eCO_2_ [ppm]	CCS811
11	TVOC [ppb]	CCS811
12	Sensor resistance [Ω]	CCS811
13	CO_2_ [ppm]	iAQ-Core
14	TVOC [ppb]	iAQ-Core
15	Sensor resistance [Ω]	iAQ-Core
16	Clean air/Sample	
17	Warning	

^1^ Raw data. Concentration can be computed from measurement with a reference concentration.

**Table 3:** Confusion matrix obtained in leave-one-out cross-validation (LOOCV).

	NO_2_ 40 µg/m^3^	NO_2_ 80 µg/m^3^	NO_2_ 120 µg/m^3^	NO_2_ 165 µg/m^3^	NO_2_ 205 µg/m^3^	NO 7.7 µg/m^3^	NO 15.5 µg/m^3^	NO 38.5 µg/m^3^	NO 77 µg/m^3^
**NO_2_ 40 µg/m^3^**	9	0	0	0	0	0	0	0	0
**NO_2_ 80 µg/m^3^**	0	9	0	0	0	0	0	0	0
**NO_2_ 120 µg/m^3^**	0	0	8	1	0	0	0	0	0
**NO_2_ 165 µg/m^3^**	0	0	1	6	2	0	0	0	0
**NO_2_ 205 µg/m^3^**	0	0	0	1	7	0	1	0	0
**NO 7.7 µg/m^3^**	0	0	0	0	0	9	0	0	0
**NO 15.5 µg/m^3^**	0	0	0	0	0	0	9	0	0
**NO 38.5 µg/m^3^**	0	0	0	0	0	0	0	9	0
**NO 77 µg/m^3^**	0	0	0	0	0	0	0	0	9

**Table 4:** Estimated LOD (µg/m^3^).

	BME680	SGP30_1	SGP30_2	CCS811	iAQCore
**NO_2_**	40.44	38.64	18.87	15.28	48.04
**NO**	2.3	2.52	1.60	4.47	25.75

## References

[1] WHO 9 Out of 10 People Worldwide Breathe Polluted Air, but More Countries are Taking Action. https://www.who.int/news-room/detail/02-05-2018-9-out-of-10-people-worldwide-breathe-polluted-air-but-more-countries-are-taking-action.

[2] Guan W.-J., Zheng X.-Y., Chung K.F., Zhong N.-S. (2016). Impact of air pollution on the burden of chronic respiratory diseases in China: time for urgent action. Lancet.

[3] Kim K.E., Cho D., Park H.J. (2016). Air pollution and skin diseases: Adverse effects of airborne particulate matter on various skin diseases. Life Sci..

[4] Arden Pope C. (2015). Air Pollution and Cardiovascular Disease. Curr. Probl. Cardiol..

[5] McKercher G.R., Salmond J.A., Vanos J.K. (2017). Characteristics and applications of small, portable gaseous air pollution monitors. Environ. Pollut..

[6] Arroyo P., Lozano J., Suárez J. (2018). Evolution of Wireless Sensor Network for Air Quality Measurements. Electronics.

[7] Mead M.I., Popoola O.A.M., Stewart G.B., Landshoff P., Calleja M., Hayes M., Baldovi J.J., McLeod M.W., Hodgson T.F., Dicks J. (2013). The use of electrochemical sensors for monitoring urban air quality in low-cost, high-density networks. Atmos. Environ..

[8] Huang Y., Hu L., Yang D., Liu H. (2017). Air-Sense: indoor environment monitoring evaluation system based on ZigBee network. IOP Conf. Ser. Earth Environ. Sci..

[9] Spinelle L., Gerboles M., Kok G., Persijn S., Sauerwald T. (2017). Review of portable and low-cost sensors for the ambient air monitoring of benzene and other volatile organic compounds. Sensors.

[10] Schneider P., Castell N., Vogt M., Dauge F.R., Lahoz W.A., Bartonova A. (2017). Mapping urban air quality in near real-time using observations from low-cost sensors and model information. Environ. Int..

[11] Lung C., Jones R., Zellweger C., Karppinen A., Penza M., Dye T., Hüglin C., Ning Z., Leigh R., Hagan D., Lewis A.C., Von Schneidemesser E., Peltier R. (2018). Low-Cost Sensors for the Measurement of Atmospheric Composition: Overview of Topic and Future Applications (WMO).

[12] Rawat P., Singh K.D., Chaouchi H., Bonnin J.M. (2014). Wireless sensor networks: A survey on recent developments and potential synergies. J. Supercomput..

[13] Laport-López F., Serrano E., Bajo J., Campbell A.T. (2019). A review of mobile sensing systems, applications, and opportunities. Knowl. Inf. Syst..

[14] Khan W.Z., Xiang Y., Aalsalem M.Y., Arshad Q. (2013). Mobile phone sensing systems: A survey. IEEE Commun. Surv. Tutor..

[15] Macias E., Suarez A., Lloret J. (2013). Mobile sensing systems. Sensors.

[16] Park S.Y., Kim Y., Kim T., Eom T.H., Kim S.Y., Jang H.W. (2019). Chemoresistive materials for electronic nose: Progress, perspectives, and challenges. InfoMat.

[17] Borrego C., Costa A.M., Ginja J., Amorim M., Coutinho M., Karatzas K., Sioumis T., Katsifarakis N., Konstantinidis K., De Vito S. (2016). Assessment of air quality microsensors versus reference methods: The EuNetAir joint exercise. Atmos. Environ..

[18] Barsan N., Koziej D., Weimar U. (2007). Metal oxide-based gas sensor research: How to?. Sens. Actuators B Chem..

[19] Catini A., Papale L., Capuano R., Pasqualetti V., Di Giuseppe D., Brizzolara S., Tonutti P., Di Natale C. (2019). Development of a sensor node for remote monitoring of plants. Sensors.

[20] Yurko G., Roostaei J., Dittrich T., Xu L., Ewing M., Zhang Y., Shreve G. (2019). Real-Time Sensor Response Characteristics of 3 Commercial Metal Oxide Sensors for Detection of BTEX and Chlorinated Aliphatic Hydrocarbon Organic Vapors. Chemosensors.

[21] Rüffer D., Hoehne F., Bühler J. (2018). New digital metal-oxide (MOx) sensor platform. Sensors.

[22] Omidvarborna H., Kumar A., Kim D.-S. (2015). NOx emissions from low-temperature combustion of biodiesel made of various feedstocks and blends. Fuel Process. Technol..

[23] De Vito S., Esposito E., Salvato M., Popoola O., Formisano F., Jones R., Di Francia G. (2018). Calibrating chemical multisensory devices for real world applications: An in-depth comparison of quantitative machine learning approaches. Sens. Actuators B Chem..

[24] Gutierrez-Osuna R. (2002). Pattern analysis for machine olfaction: A review. IEEE Sens. J..

[25] Marco S., Gutiérrez-Gálvez A. (2012). Signal and Data Processing for Machine Olfaction and Chemical Sensing: A Review. IEEE Sens. J..

[26] Gas Sensor BME680 | Bosch Sensortec. https://www.bosch-sensortec.com/products/environmental-sensors/gas-sensors-bme680/.

[27] Multi-Pixel Gas Sensors SGP | Sensirion. https://www.sensirion.com/en/environmental-sensors/gas-sensors/multi-pixel-gas-sensors/.

[28] CCS811 | ams. https://ams.com/ccs811.

[29] iAQ-Core C | ams. https://ams.com/iaq-core-c#tab/features.

[30] Rahman M.A., Asyhari A.T. (2019). The emergence of internet of things (Iot): Connecting anything, anywhere. Computers.

[31] Arroyo P., Herrero J., Suárez J., Lozano J., Arroyo P., Herrero J.L., Suárez J.I., Lozano J. (2019). Wireless Sensor Network Combined with Cloud Computing for Air Quality Monitoring. Sensors.

[32] European Comission Standards—Air Quality—Environment—European Commission. https://ec.europa.eu/environment/air/quality/standards.htm.

[33] Esbensen K.H., Geladi P. (2009). Principal Component Analysis: Concept, Geometrical Interpretation, Mathematical Background, Algorithms, History, Practice. Compr. Chemom..

[34] Gardner M.W., Dorling S.R. (1998). Artificial neural networks (the multilayer perceptron)—A review of applications in the atmospheric sciences. Atmos. Environ..

[35] Currie L.A. (1995). Nomenclature in evaluation of analytical methods including detection and quantification capabilities (IUPAC Recommendations 1995). Pure Appl. Chem.

[36] Burgués J., Hernández V., Lilienthal A.J., Marco S. (2019). Smelling nano aerial vehicle for gas source localization and mapping. Sensors.

